# The Damage-Associated Molecular Patterns (DAMPs) as Potential Targets to Treat Osteoarthritis: Perspectives From a Review of the Literature

**DOI:** 10.3389/fmed.2020.607186

**Published:** 2021-01-18

**Authors:** Cécile Lambert, Jérémie Zappia, Christelle Sanchez, Antoine Florin, Jean-Emile Dubuc, Yves Henrotin

**Affiliations:** ^1^MusculoSKeletal Innovative Research Lab, University of Liège, Institute of Pathology, CHU Sart-Tilman, Liège, Belgium; ^2^Orthopaedic Department, University Clinics St. Luc, Brussels, Belgium; ^3^Physical Therapy and Rehabilitation Department, Princess Paola Hospital, Vivalia, Marche-en-Famenne, Belgium

**Keywords:** osteoarthritis, cartilage, immunity, inflammation, synovitis

## Abstract

During the osteoarthritis (OA) process, activation of immune systems, whether innate or adaptive, is strongly associated with low-grade systemic inflammation. This process is initiated and driven in the synovial membrane, especially by synovium cells, themselves previously activated by damage-associated molecular patterns (DAMPs) released during cartilage degradation. These fragments exert their biological activities through pattern recognition receptors (PRRs) that, as a consequence, induce the activation of signaling pathways and beyond the release of inflammatory mediators, the latter contributing to the vicious cycle between cartilage and synovial membrane. The primary endpoint of this review is to provide the reader with an overview of these many molecules categorized as DAMPs and the contribution of the latter to the pathophysiology of OA. We will also discuss the different strategies to control their effects. We are convinced that a better understanding of DAMPs, their receptors, and associated pathological mechanisms represents a decisive issue for degenerative joint diseases such as OA.

## Introduction

Osteoarthritis (OA) is the most common joint disease affecting more than 70 million people across the United States (*CDC: Arthritis: At a Glance*) and Europe (1). As underlined by many authors, it has long been considered as “a wear and tear disease” of cartilage associated with age, it is in reality a complex disorder affecting the “whole joint” (2) and the pro-inflammatory pathways of immunity that can culminate in illness (3–5).

**Figure 1:**
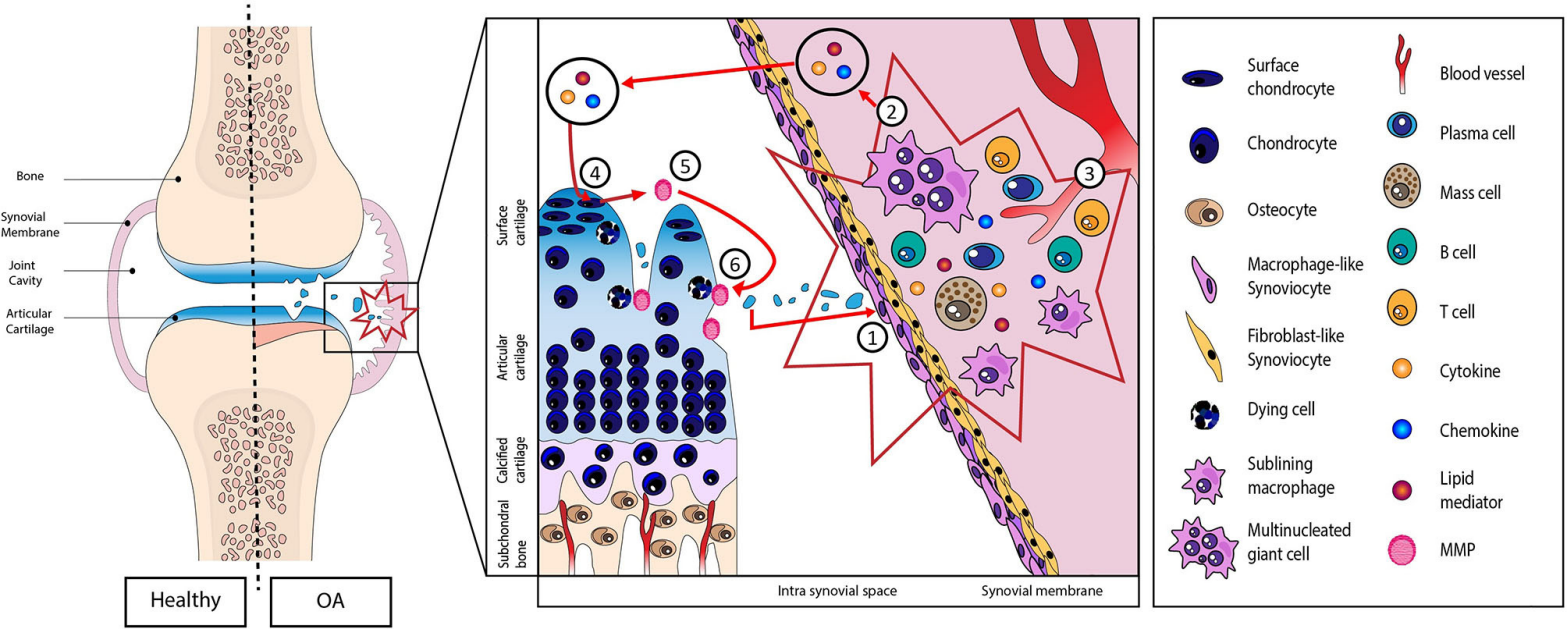
Schematic representation of the role of damage-associated molecular patterns (DAMPs) in the initiation and perpetuation of the low-grade systemic inflammation. (1) DAMPs released from extracellular matrix to the joint cavity during cartilage degradation. (2) Proliferation and hyperplasia of the lining cells along with inflammatory cell infiltration and (3) neoangiogenesis. (4) Production of inflammatory mediators (cytokines, chemokines, lipid mediators, and DAMPs themselves) into the synovial fluid. (5) These mediators then activate chondrocytes that in turn produce metalloproteinase resulting in a vicious cycle (6) between cartilage and synovial membrane.

During the osteoarthritis (OA) process, activation of immune systems, whether innate or adaptive, is strongly associated with low-grade systemic inflammation (4, 6–10) (Figure 1). This process was initiated and driven in the synovial membrane, especially by damage-associated molecular patterns (DAMPs) released from the extracellular matrix (ECM) to the joint cavity during cartilage degradation (4, 11–13). Briefly, these fragments released into the synovial cavity stimulate the production and release of inflammatory mediators (cytokines, chemokines, lipid mediators, and DAMPs themselves) by the synovial cells (macrophages and fibroblasts) into the synovial fluid. These mediators, in turn, activate chondrocytes that produce metalloproteinase, resulting in a vicious cycle between cartilage and synovial membrane (12).

DAMPs are defined as endogenous stimuli that are released either from ECM or from dying cells (14). “*Intracellular*” DAMPs consist of a set of immunogenic molecules released from the breakdown of necrotic and apoptotic cells such as calcium-binding protein S-100, high-mobility group box protein 1 (HMGB1), or uric acid, while “*extracellular*” DAMPs correspond to the ECM components (glycoproteins, proteoglycans, or glycosaminoglycans). The biological activity of these DAMPs goes through pattern recognitions receptors (PRRs) including Toll-like receptors (TLRs), NOD-like receptors (NLRs), and Receptor for Advanced Glycosylation End products (RAGEs) (15). These PRRs have been identified, notably, on the surface of immune cells, chondrocytes, osteoblasts, and synoviocytes. The binding of DAMPs to these receptors initiates downstream signaling cascades leading to the activation of several transcription factors, such as notably, the nuclear factor-κB (NF-κB), an inflammatory response key regulator (16). This activation leads to the release of various factors like catabolic factors [matrix metalloproteinase (MMP)-1,−3,−9, and−13], cytokines [tumor necrosis factor (TNF)-α, interleukin (IL)-1β, and IL-6], chemokines [C-C motif chemokine ligand (CCL)-2,−5,−7,−8], cathepsins (B, K, and L), and complement cascade (17), factors described as essential in OA pathogenesis.

The aim of this review is to focus on the roles of DAMPs in the pathogenesis of OA. We have also researched the ways to block DAMP activity and summarized the current therapeutic approaches targeting DAMPs activity.

In this context, the literature search was performed using the Pubmed/Medline database between January 2010 and April 2020. All original papers, systematic and narrative reviews, were included. Searches were performed using the search terms “osteoarthritis,” “cartilage,” “synovium,” “DAMP,” and “immunity.” Papers published in English and reporting on the search criteria were included in this manuscript, while duplicates were removed from the selection. As a consequence, 98 articles were analyzed, and their relevant data were included in this narrative review.

## Extracellular Damage-Associated Molecular Patterns From Cartilage Extracellular Matrix

Current evidences indicate that endogenous molecular products derived from ECM disruption can function as DAMPs to activate PRRs (14, 18). MMPs and/or aggrecanases [a disintegrin and metalloproteinase with thrombospondin motifs (ADAMTS)-4 and−5] are able to cleave a large number of ECM molecules (Table 1), leading to the exposure of cryptic epitopes and recognition with ligand receptors (18). Inflammatory mediators produced may in turn stimulate the production of cartilage-degrading enzymes and recruitment of inflammatory cells, thus establishing a vicious cycle between cartilage and synovial membrane that contributes to OA progression.

**Table 1:** Overview of DAMPs and their implications in the OA pathogenesis.

**DAMPs**	**Receptors**	**Activated signaling pathway**	**Biological effects**	**Species**	**References**
**Extracellular DAMPs**					
Fibronectin fragments	TLR-2	MyD88	- ↑ catabolic cytokines - ↑ MMPs •Suppression proteoglycan synthesis	Human	(19)
	/	IL-1ra	- ↑ TLR-2 expression	Human	(20, 21)
Hyaluronan (Low molecular weight)	TLR-2;−4	CD44 and MyD88	- NO and MMP production - Dendritic cell and macrophage activation	Human, Mouse	(22, 24)
	NLRP3		- IL-1 release	Mouse	(25)
Tenascin-C	TLR-4		- Cytokine synthesis (TNF-α, IL-6,−8)	Human	(27, 28)
Lubricin	TLR-2,-4,-5		- Anti-inflammatory effect (↓ cytokine expression)	Human, Rat	(30)
Fibromodulin, Osteoadherin Chondroadherin	C1q	Classical pathway	- MAC upregulation	Human	(33)
Biglycan	TLR-2,−4	P38, ERK and NF-κB	- ↑ TNF-α and MIP-2 expression - ↑TLR-4 expression - ↑ Catabolic factor expression - ↓ Matrix component expression	Human, Mouse	(34–36)
	C1q	Classical pathway	- Inhibitory function on the classical pathway	Human	(37)
Fibronectin	TLR-4	P38 and NF-κB	- Cytokine release from mast cells and T cells	Mouse, Human	(39–41)
	TLR-2	MyD88	- Catabolic responses (MMP-3 upregulation, cleavage of fibronectin, or type II collagen)	Human	(20)
Native Type II collagen	DDR2	P38 and NF-κB	- Cytokine and MMP induction	Human	(42)
N-terminal telopeptide of collagen type II (29-mer)	/	Protein kinase C and p38	- ↑Cathepsins B, L, and K - ↑MMP-2,−3,−9, and -13	Human, Bovine	(43, 44)
24-mer synthetic peptide of type II collagen (CB12-II)	/	PI3K/Akt and NF-κB	- MMP-13 induction	Bovine, Human	(45, 46)
Coll2-1	TLR-4	NF-κB	- ↑ IL-8 - ↑ MMP-3	Human	(47)
Aggrecan 32-mer fragment	TLR-2	NF-κB	- ↑ Protease expression (MMP-13 and ADAMTS-5) - ↓ Col2A1 and aggrecan expression	Mouse, Human	(48)
Collagen IX (NC4)	C4, C3, and C9		- Inhibition of complement activation	Human	(49)
COMP	C3b	Alternative pathway	- Activation complement system	Human	(50)
	C1Q	Classical and lectin pathways	- Inhibition complement system	Human	(50)
**Intracellular DAMPs**					
Gc-globulin, α1-microglobulin, α2-macroglobulin	TLR-4		- ↑Inflammatory cytokine and growth factor production	Human	(56)
Fibrinogen	TLR-4	NF-κB	- ↑ Chemokine production	Mouse, Human	(58–60)
			Attraction of T cells, neutrophils, and additional macrophages		
S100A8/S100A9	TLR-4	NF-κB	- ↓ Anabolic factor production - ↑Catabolic factor production Osteophyte formation Synovitis - ↑ Knee symptoms, cartilage defects, and MMP-3 serum levels	Mouse, Human	(61–64)
AIIt	TLR-4	MAPK and NF-κB	- Macrophage activation - Inflammatory mediator secretion	Human	(65, 66)
S100A12	RAGE	p38 and NF-κB	- ↑ MMP-13 and VEGF expression and release	Human	(67)
HMGB1	RAGE, TLR-2,-4	ERK and NF-κB	- Promotes chemotaxis - ↑ Cytokines, chemokines, and MMP expression	Human	(71–74)
CPPD, BCP	TLR-2, NRLP3	MAPK, NF-κB	- ↑MMPs, prostaglandin and inflammatory cytokine production	Mouse, Human, Bovine	(77–80)
			- NO production		
			- Neutrophil apoptosis inhibition		

*OA, osteoarthritis; DAMPs, damage-associated molecular patterns; ADAMTS, A Disintegrin and Metalloproteinase with Thrombospondin Motifs; HMGB1, high-mobility group box protein 1; TLRs, Toll-like receptors; RAGEs, Receptor for Advanced Glycosylation End products; NF-κB, nuclear factor-κB; MMP, matrix metalloproteinase; TNF, tumor necrosis factor; IL, interleukin; HA, hyaluronan; NO, nitric oxide; MyD88, myeloid differentiation primary response 88; NLRP3, NOD-like receptor family, pyrin domain containing 3; MAC, membrane attack complex; ERK, extracellular signal-regulated kinase; Col2A1, collagen type II alpha 1 chain; NC4, noncollagenous domain 4; COMP, cartilage oligomeric matrix protein; BSP-1, bone sialoprotein 1; SIBLINGs, small integrin-binding ligand N-linked glycoproteins; VEGF, vascular endothelial growth factor; S100A8, S100 calcium-binding protein A8; S100A9, S100 calcium-binding protein A9; S100A12, S100 calcium-binding protein A12; CPPD, calcium pyrophosphate deposition; BCP, basic calcium phosphate; PI3K, phosphoinositide 3-kinase*.

Homandberg and Hui (19) suggested that ECM breakdown fragments may promote inflammation and cartilage loss. So, during cartilage degradation, proteolytic cleavage of fibronectin (Fn) generates fibronectin fragments with cartilage chondrolytic activities. These are exercised through the increase of MMP expression, the suppression of proteoglycan synthesis, or the increase of cytokines. They highlighted that an amino-terminal 29-KDa fibronectin fragment (Fn-f) was able to induce, in human articular cartilage explant cultures, the production of not only pro-inflammatory cytokines, such as TNF-α, IL-1β, and IL-1α, but also MMPs, MMP-1 and−3. In human chondrocytes, Hwang et al. (20) also demonstrated that Fn-f was able to regulate cartilage catabolism through TLR-2. Furthermore, Fn-f is also able to upregulate TLR-2 expression through IL-1ra, suggesting an autocrine/paracrine regulation of IL-1 activity (21).

Hyaluronan (HA) can be described as a non-sulfated component of the ECM, commonly and abundantly found in the synovial fluid. Exogenous HA is injected in knee joints with the aim to treat joint inflammation through a mechanical effect leading to the inhibition of inflammatory pathways, stimulation of cartilage anabolism, and reduction of free radical production (22). However, HA action seems related to its molecular mass, HA of high molecular weight being anti-inflammatory and inversely for low-molecular-weight HA (23). In this context, low-molecular-weight HA, resulting from the HA degradation at sites of inflammation and tissue injury, induced nitric oxide (NO) and MMP production by mechanisms dependent on CD44 and myeloid differentiation factor 88 (MyD88) through TLR-2,−4 (22). The fragmentation products of hyaluronic acid containing sugar units of 4–16 oligosaccharide size have also been demonstrated to act as potent activators of dendritic cells and macrophages *via* TLR-4 (24). Yamasaki et al. (25) also demonstrated that small HA oligosaccharides activate inflammasome through NOD-like receptor family, pyrin domain containing 3 (NLRP3) and release of IL-1β.

Tenascin-C (TN-C) belongs to the ECM glycoprotein family. It is involved in tissue injury and repair. In OA, its expression is upregulated in cartilage and synovium. TN-C is also elevated in OA synovial fluid when compared to healthy one. Sofat et al. (26) demonstrated that TN-C fragments [the epidermal growth factor-like (EGF-L) and Fn type III domains 3–8 of TN-C] contributes to cartilage matrix degradation by inducing aggrecanase activity. Recently, Midwood et al. (27) also highlighted that TN-C induces cytokine production (TNF-α, IL-6, and IL-8) through the activation of TLR-4 in human macrophages and synovial fibroblasts. Zuliani-Alvarez et al. (28) have identified three distinct sites within the C-terminal fibrinogen-like globe (FBG) domain of TN-C contributing to TLR-4 activation.

Lubricin/proteoglycan 4 (PRG4) is a mucin-like glycoprotein. It is present at the surface of articular cartilage and contributes to the maintenance and integrity of the joint. Decreased expression of PRG4 is associated with OA progression (29). However, recently, Iqbal et al. (30) demonstrated in synovial cells that the full-length recombinant human PRG4 can regulate the immune response *via* TLRs (TLR-2,−4, and−5) and, therefore, modifies cytokine and chemokine secretion. Thus, the PRG4/TLR binding activating the NF-κB pathway is involved in maintaining the homeostatic state of the cell. However, when TLR-2,−4, or−5 is bound to another agonist, in turn, PRG4 activates inflammatory responses *via* an alternative pathway that does not appear to be nuclear factor NF-κB dependent (30).

Decorin, biglycan, fibromodulin, lumican, PRELP (proline-arginine-rich-end-leucine-rich repeat protein), chondroadherin, and osteoadherin are members of the small leucine-rich repeat protein (SLRP) family, as reviewed by Zappia et al. (31). Fibromodulin is a keratan sulfate proteoglycan found in cartilage and tendon. Sjöberg et al. (32) showed that fibromodulin triggers complement activation. Sjöberg et al. (33) revealed that fibromodulin upregulated the membrane attack complex (MAC) from human OA sera. In addition, these authors also demonstrated that osteoadherin and chondroadherin, like fibromodulin binds C1q and activates classical pathway (33). In macrophages, biglycan, a small leucine-rich proteoglycan, has been demonstrated to act as an endogenous ligand of TLR-4 and TLR-2. This binding results in a rapid activation of p38, extracellular signal-regulated kinase (ERK), and NF-κB and, subsequently, the stimulation of TNF-α and macrophage inflammatory protein-2 (MIP-2) expression (34). Barreto et al. (35) also demonstrated that soluble biglycan is commonly detected in knee synovial fluid of patients with advanced knee OA or rheumatoid arthritis (RA). Soluble biglycan upregulates TLR-4 expression in human OA chondrocytes, increases both expression and concentrations of catabolic factors (ADAMTS-4, ADAMTS-5, MMPs, NO, cathepsin K, IL-6, and IL-8), and decreases the expression of matrix components (collagen type II, aggrecan), globally resulting in net loss of cartilage (35). Recently, Avenoso et al. (36) also reported that human chondrocytes treated with biglycan produces several inflammatory mediators (IL-1β, IL-6, MMP-13, and IL-17) and activates NF-κB and TLR-4 (36). Conversely, biglycan and decorin can also bind to C1q and then inhibit the classical pathway (37).

Fn, whose fragments were found increased in OA cartilage and synovial fluid, was also identified as an activator of TLR (38). Two Fn domains have been identified as TLR activators: the extra Type III domain and FnEDA. These domains stimulate TLR-4-dependent cytokine release from mast cells and T cells (39, 40). Kelsh et al. (41) also identified NF-κB and p38 signaling pathways as transducers of Fn-f/TLR signals. Hwang et al. (20) demonstrated in human chondrocytes the probable involvement of MyD88-dependent TLR-2 signaling pathway in Fn fragment release and mediated cartilage catabolic responses.

Type II collagen-derived peptides also seem to act as potent activators of innate immunity. In human chondrocytes, Klatt et al. (42) have observed the collagen II-dependent induction of both cytokines (IL-1β,−6, and−8) and MMPs (MMP-1,−3,−13, and−14) involved in p38 and NF-κB signaling. In human articular chondrocytes, an N-terminal fragment of type II collagen (29-mer fragment) stimulated the production of cathepsins B, L, and K through the activation of protein kinase C and p38 mitogen-activated protein kinase (MAPK) (43). Fichter et al. (44) demonstrated that mRNA and protein levels of MMP-2,−3,−9, and−13 were also upregulated by this 29-mer peptide. In a cartilage explant culture model, Tchetina et al. (45) reported that a 24-mer synthetic peptide of type II collagen (named CB12-II) was able to stimulate type II collagen cleavage through MMP-13 induction. Subsequently, in a study conducted in human OA chondrocytes, Yasuda (46) demonstrated that CB12-II stimulated phosphoinositide 3-kinase (PI3K)/Akt, leading to NF-κB activation. Recently, our team demonstrated that Coll2-1, a synthetic peptide located in the triple helical part of the type II collagen molecule and currently used as a biomarker of cartilage degradation, activates synoviocytes to produce IL-8 and chondrocytes to produce MMP-3. We also demonstrated that these Coll2-1 effects were mediated through TLR-4 and NF-κB signaling pathway activation (47).

Lees et al. (48) also examined the bio-activity of an aggrecan 32-mer fragment. They reported that it increased MMP-13 and ADAMTS-5 mRNA expression and decreased Col2A1 and aggrecan mRNA through TLR-2- and NF-κB-dependent signaling.

Type IX Collagen is located at the surface of fibrils formed by collagen II, playing roles in tissue stability and integrity. Collagen IX cleavage and loss of the N-terminal non-collagenous domain 4 (NC4) precede major damage of collagen II fibrils and can therefore be considered as key early steps in cartilage degradation. Kalchishkova et al. (49) showed that NC4 is able to bind C4, C3, and C9 and to directly inhibit C9 polymerization and MAC formation and can therefore be considered as a complement system inhibitor. NC4 interactions with fibromodulin and osteoadherin also inhibited complement activation by these proteins (49).

The cartilage oligomeric matrix protein (COMP), detected with abnormally high levels in OA synovial fluid, can also fix the complement system *via* C3b and C9 through an alternative complement pathway. COMP is also able to inhibit classical and lectin pathways through its interaction with C1q and mannose-binding lectin (50). The same observation is reported with cartilage fragments decorin and biglycan (51).

The bone sialoprotein I (BSP-1) is described as a non-collagenous ECM protein, member of the small integrin-binding ligand N-linked glycoproteins (SIBLINGs) family, expressed by many cell types among which are osteoblasts, osteoclasts, chondrocytes, synoviocytes, macrophages, and activated T cells (52). BSP-1 levels are increased in OA joint (synovial fluid and articular cartilage) compared to healthy controls, and these levels are correlated with the severity of joint lesion and the inflammatory status of patients (53). Furthermore, elevated levels of BSP-1 activate both an increase of MMP-13 expression and NF-κB activation and, consequently, the increased production of cytokines and chemokines, leading to NO, prostaglandin E2 (PGE_2_), IL-6, and IL-8 production and imbalance the cartilage homeostasis (54). Moreover, BSP regulates T cell development, increases Th1 differentiation, suppresses Th2, and supports Th17 differentiation. Tardelli et al. (55) also demonstrated that BSP-1 has a key role not only in monocyte chemotaxis and macrophage differentiation but also in 4 macrophage proliferation.

## Intracellular Damage-Associated Molecular Patterns

### Plasma Proteins

Sohn et al. (56) have recently identified by mass spectrometry in synovial fluid three plasma proteins of interest: Gc-globulin (vitamin D-binding protein), α1-microglobulin, and α2-macroglobulin. They showed that these plasma proteins induced TLR-4-dependent production of a large number of inflammatory cytokines and growth factors like IL-1β, IL-6, TNF-α, and vascular endothelial growth factor (VEGF). Fibrinogen, also found with increased levels in OA synovial fluid (57) and whose amount of fibrin deposited in the synovial membrane positively correlates with the severity of OA, is able to stimulate the production of chemokines [IL-8, monocyte chemoattractant protein (MCP)-1, …] by macrophages in a TLR-4-dependent manner, promoting attraction of T cells, neutrophils, and additional macrophages (58–60).

### Alarmins

Large amounts of S100A8 and its binding partner S100A9 are released by neutrophils, monocytes, and activated macrophages. This heterodimer is highly expressed by synovial tissue in experimental OA models and involved in synovitis and cartilage destruction. Furthermore, high levels may predict joint destruction in humans (61). Recently, in human OA tissue, Schelbergen et al. (62) also demonstrated that S100A8/S100A9 levels were closely associated with cartilage loss and that they stimulate chondrocytes to produce more MMPs and cytokines (catabolic factors) but less type II collagen and aggrecan (anabolic factors). This effect was triggered by TLR-4. These authors also highlighted the role of S100A8/S100A9 in osteophyte formation and synovial activation in collagenase-induced OA and destabilized medial meniscus OA (62). In a study conducted in patients with knee OA, Ruan et al. (63) also demonstrated the association between serum levels S100A8/S100A9 and increased knee symptoms, cartilage defects, and MMP-3 serum levels. Finally, the canonical Wnt signaling pathway plays a key role in S100A8/S100A9 complex activity (64).

S100A10 forms with annexin II, a heterotetrameric complex called AIIt. This last activates human macrophages, which in turn secretes a number of inflammatory mediators including TNF *via* TLR-4 (65). Moreover, Song et al. (66) also demonstrated that the production of cytokines (TNF, IL-1β, and IL-10) in human chondrocytes was dependent on S100A10 through MAPK and NF-κB pathways.

Recently, S100A12 expression was found to be increased in OA cartilage and to contribute to the development of OA through an increase of MMP-13 and VEGF expression resulting from p38 MAPK and NF-κB pathway activation (67). Wang et al. (68) has also demonstrated that S100A12 levels in synovial fluid may correlate to clinical severity of patients with primary knee OA. In OA synovial fluid, S100A12 is significantly overexpressed, and Meijer et al. (69) highlighted this role in the innate and acquired inflammatory responses. This role in this innate immunity would be linked to RAGE receptors (70).

HMGB1 is released by necrotic cells or secreted by macrophages and other myeloid cells in response to inflammatory cytokines (IL-1β and TNF). Magna et al. (71) highlighted its role as alarmin binding to a lot of receptors, cytokines, and chemokines to stimulate the innate immune system. Since then, through cytokine production *via* TLR-4, HMGB1 promotes chemotaxis. HMGB1 was found overexpressed in the synovial fluid and cartilage of OA patients (72, 73). Thus, several authors reported that HMGB1 and RAGE are expressed in OA cartilage, and the activation of OA chondrocytes triggers ERK and NF-κB phosphorylation as well as MMP expression. García-Arnandis et al. (74) also reported that in OA synoviocytes, HMGB1 cooperates with IL-1β to amplify the inflammatory response resulting in the production of cytokines, chemokines, and MMPs. It can also trigger and prolong inflammatory responses *via* TLR-2,−4 but also RAGE.

### Crystals

Microcrystals associated with joint diseases trigger inflammation and beyond innate immunity responses through both inflammasome-dependent and inflammasome-independent pathways (75, 76). Rosenthal (77) highlighted that calcium-containing crystals [calcium pyrophosphate dehydrate (CPPD) and basic calcium phosphate (BCP)] contribute to OA pathogenesis. Thus, these crystals exert direct effects both on synoviocytes and chondrocytes through the production of MMPs, prostaglandins, and inflammatory cytokines and this, *via* NF-κB, MAPK signals, and NO-dependent pathways. Furthermore, these crystals, combined with uric acid presence, are also able to interact with NLRP3 (78, 79) and subsequent IL-1β and IL-18 activation. Liu-Bryan et al. (80) showed also that CPPD crystals induced NO production in a TLR-2-dependent manner. Rosenthal (77) also report that these calcium-containing crystals directly affect inflammatory cells. For example, CPPD crystals can inhibit neutrophil apoptosis and extend the inflammatory response.

## Cellular Receptors Involved in Damage-Associated Molecular Patterns Activity

DAMPs exert their biological activities through receptors TLR, NLR, and RAGE. Actually, 10 functional TLRs were identified in humans numbered TLR1–10. TLR-1,−2,−4,−5,−6, and−10 are located at the cell surface, while TLR-3,−7,−8, and−9 are present at the endolysosomal membrane (81). The signaling pathways activated by TLR involve the recruitment of adapter proteins such as MyD88, TIR domain-containing adaptor-inducing interferon (TRIF), TRIF-related adaptor molecule (TRAM), MyD88-adaptor like (Mal), and the activation of nuclear factors among which NF-κB. TLR also initiates distinct parallel signaling pathways leading to MAPK and PI3K activation (82). These latter regulate the transcription, mRNA stability, and translation of pro-inflammatory cytokine genes (TNF-α, IL-1β, or IL-6) and cell membrane-bound co-stimulatory molecules [intercellular adhesion molecule (ICAM)-1]. TLR-2 and−4 play a key role in OA pathogenesis since their expressions were demonstrated to be increased particularly at sites of cartilage lesions and inflammatory synovial membranes (83, 84). TLR-4 is expressed by numerous cell types in the joint including immune cells, chondrocytes, osteoblasts, and synoviocytes (83). Activation of TLR-4 leads to upregulation of IL-1β, MMP expression, NO release, and PGE_2_ synthesis, as well as downregulation of aggrecan core protein and type II collagen synthsesis (84, 85). Recently, comparing human cartilage from carpometacarpal (CMC)-I and knee joints, Barreto et al. (86) have observed that TLRs, and specially TLR-4, are differentially expressed depending on cartilage origin. Soluble forms of TLR-2 and−4 were also detected in the OA synovial fluid with sTLR-4 being elevated in OA knee comparing to healthy knee. Studies also highlighted that TLR1–7 and−9 expression was upregulated in the synovium of OA patients. Increased concentrations of several DAMPs (Fn, HA, Tn-C, PRG4, biglycan, or S100 family) are found in the OA synovial joint fluids and tissues and are able to activate TLRs; among them, Fn, HA, Tn-C, PRG4, biglycan, or S100 family.

NLRs are intracellular sensors of pathogen-associated or endogenous danger-associated molecular patterns (87). NLR system counts 22 cytoplasmic proteins including the nucleotide-binding oligomerization domains (NOD) and Nacht domain-containing, leucine-rich repeat-containing and pyrin domain-containing protein (NALP) subfamilies. The best characterized NLR is NLRP3, highly expressed in macrophages, chondrocytes, synoviocytes, and osteoblasts (76). Once activated, NLRP3 forms an oligomer able to interact with adapter proteins, C-terminal caspase recruitment domain (ASC), and Cardinal, creating a complex able to recruit procaspase-1. In turn, it is activated and the result is a multimeric structure named “the inflammasome,” which is capable of inducing maturation and secretion of pro-inflammatory cytokines (such as IL-1β, IL-1α, IL-18) (88, 89). In OA, NLRP3 has been associated with crystal-induced inflammation triggered by uric acid, calcium pyrophosphate, and hydroxyapatite crystals (76). These microcrystals are interpreted as DAMPs by the innate immune system and cause inflammation (75).

RAGE, a transmembrane receptor, which belongs to the immunoglobulin gene superfamily (90), is also bound by DAMPs. RAGE is composed of three distinct regions including an extracellular region responsible for ligand interaction through its V domain, a transmembrane domain, and a cytoplasmic domain responsible for downstream signaling. Activation of RAGE leads to the activation of NF-κB and MAPK pathways, which themselves induce the expression of pro-inflammatory and catabolic genes. Initially identified as a receptor for advanced glycation end-products (AGEs), it can also be bound by several DAMPs including HMGB1, S100 proteins, or amyloid-β protein (90, 91).

## Damage-Associated Molecular Patterns, Perspectives, and Target Therapeutics

Several strategies have been suggested especially to control TLR-4 signaling. TLR-4 signaling activities may be downregulated by agonist blockers, activators of antagonist pathways, or new molecules. Among the agonist blockers, high-molecular-weight hyaluronic acid acts as a dressing blocking TLR access to short HA oligosaccharides (HA 4-mers) (92). Another agonist is the blocking peptide, Pep-1. The latter, a 12-mer peptide, inhibits low-molecular-weight HA binding to TLR-4. In a mouse chondrocyte model, Campo et al. (93) hypothesize that hydrophobic and/or polar residues of Pep-1 function as primary binding sites to HA, therefore reducing its binding to TLR-4 and subsequently the pro-inflammatory responses associated with TLR-4 activation.

Another strategy is the activation of antagonist pathways. Among these, peroxisome proliferator-activated receptor γ (PPARγ), PGD_2_, vasoactive intestinal peptide (VIP), adenosine 2A receptor (A2AR), and bone morphogenic protein 7 (BMP-7) are reported to be the most promising targets. PPARγ has been well characterized as intracellular receptor and transcription factor with anti-inflammatory functions in cartilage. In this context, molecules such as rosiglitazone and pioglitazone, defined as PPARγ agonists, have been proposed to block TLR-4 signaling pathway. Thus, the stimulation of human chondrocytes and synovial fibroblasts by rosiglitazone inhibits TLR4 activation, leading to inhibition of TLR-4-induced catabolism and inflammation mediated by serum amyloid A. Serum amyloid proteins are major acute-phase proteins, detected in OA serum and able to trigger *via* TLR-2 and−4 stimulating cytokines (IL-6, IL-8, CXCL-1) and metalloproteinase expression (94). Pioglitazone inhibits TLR-4-mediated effects of AGEs including the induction of cyclooxygenase (Cox-2), HMGB1, IL-6, and MMP-13 (95). Besides PPARγ, PGD_2_ is another candidate pathway and innate immune inhibitor. It inhibits PGE_2_-dependent induction of TLR-4 and, subsequently, the IL-6 synthesis by chondrocytes (96). Finally, VIP, a neuropeptide produced by immune cells, is also able to inhibit in OA synoviocytes TLR-4-mediated effects including pro-inflammatory responses and TLR-4 expression (97).

Among the new compounds developed to target TLR-4 in joint tissues, we can cite the promising 6-Shogoal that was demonstrated to reduce both TLR-4-mediated innate immune responses and the catabolic TLR-4 signaling pathway in mouse and human chondrocytes (98).

Among the other receptors implicated in innate immunity, the TLR-2 is another potential therapeutic target. In the collagen-induced arthritis model in mice, TLR-2 monoclonal antibody (mAb) reduced the pro-inflammatory cytokine production (IL-12 and TNF-α) as well as the development of clinical parameters (99). Alquraini et al. (100) also evaluated the binding of PRG4 with TLR-2 and−4. It appears that PRG4 binds to these two receptors, highlighting an anti-inflammatory role for PRG4 in OA synovial fluid. With promising *in vivo* effects, we can also cite RAGE and its soluble receptor, sRAGE. This last acts as a competitive inhibitor of RAGE, inhibiting downstream signaling and integrin binding (101).

Complement system can also be a therapeutic target. So, eculizumab, a humanized monoclonal antibody, is an inhibitor of terminal complement pathway (102). It binds specifically to the complement C5 protein, inhibiting the terminal complex, MAC. The effects of methylprednisolone on complement activation in patients undergoing total knee arthroplasty are currently clinically evaluated (ClinicalTrials.Gov Identifier: NCT02332616).

Another approach is to block the biological activity of DAMPs using a specific ligand. Promising examples are found in the literature. In mouse models, blockage of the pro-inflammatory effects of S100A8/A9 using an anti-carboxylate glycan antibody has also been concluding (12). Neutralizing HMGB1 antibodies or truncated HMGB1-derived A-box proteins are currently evaluated in collagen-induced arthritis rodent models (103). Targeting NLRP3 also looks promising (76). MCC950, a small-molecule chemical inhibitor, selectively inhibits activation of NLRP3 and IL-1β production by preventing NLRP3-induced ASC oligomerization (104). Finally, within our research unit, we demonstrate that Coll2-1, a synthetic peptide, is an actor of synovitis (47). Neutralized Coll2-1 with a humanized mAb may also represent an original approach in the control of OA progression.

In addition to the therapeutic aspect, the question arises as to the clinical utility of DAMPs. A lot of authors suggest the possibility that these DAMPs could be used as diagnostic and prognostic biomarkers of OA. Thus, soluble biglycan in inflammatory renal diseases, HMGB1 in systemic lupus erythematosus, or S100 proteins in several inflammatory conditions are some examples (105, 106).

## Conclusion

Numerous pieces of evidence highlight the close link between immune response and the inflammation in OA process. The DAMPs are key actors. The list of these is constantly growing and represents interesting targets for future immunotherapy by blocking DAMP activities or their receptors. A better of understanding of DAMPs, their receptors, and associated pathological mechanisms represents an issue for degenerative joint diseases such as OA.

## Author Contributions

CL, JZ, and YH contributed to drafting the manuscript. CL, CS, AF, and YH contributed to revising the manuscript content. CL, JZ, CS, AF, J-ED, and YH contributed to approving the final version of the manuscript. All authors contributed to the article and approved the submitted version.

## Conflict of Interest

The authors declare that the research was conducted in the absence of any commercial or financial relationships that could be construed as a potential conflict of interest.

## Abbreviations

OA: osteoarthritis
DAMPs: damage-associated molecular patterns
ECM: extracellular matrix
HMGB1: high-mobility group box protein 1
PRRs: pattern recognition receptors
TLRs: Toll-like receptors
NLRs: NOD-like receptors
RAGEs: Receptor for Advanced Glycosylation End products
NF-κB: nuclear factor-κB
MMP: matrix metalloproteinase
TNF: tumor necrosis factor
CCL: C-C motif chemokine ligand
ADAMTS: A Disintegrin And Metalloproteinase with Thrombospondin Motifs
Fn: fibronectin
IL: interleukin
HA: hyaluronan
NO: nitric oxide
MyD88: myeloid differentiation primary response 88
NLRP3: NOD-like receptor family, pyrin domain containing 3
TN-C: tenascin-C
EGF-L: epidermal growth factor-like
FBG: fibrinogen-like globe
PRG4: lubricin/proteoglycan 4
PRELP: proline-arginine-rich-end-leucine-rich repeat protein
SLRP: small leucine-rich repeat protein
MAC: membrane attack complex
ERK: extracellular signal-regulated kinase
RA: rheumatoid arthritis
FnEDA: fibronectin extra domain A isoform
Col2A1: collagen type II alpha 1 chain
NC4: non-collagenous domain 4
COMP: cartilage oligomeric matrix protein
BSP-1: bone sialoprotein 1
SIBLINGs: small integrin-binding ligand N-linked glycoproteins
PGE_2_: prostaglandin E2
VEGF: vascular endothelial growth factor
MCP-1: monocyte chemoattractant protein-1
S100A8: S100 calcium-binding protein A8
S100A9: S100 calcium-binding protein A9
S100A12: S100 calcium-binding protein A12
CPPD: calcium pyrophosphate deposition
BCP: basic calcium phosphate
TRIF: TIR domain-containing adaptor-inducing interferon
TRAM: TRIF-related adaptor molecule
Mal: MyD88-adaptor like
PI3K: phosphoinositide 3-kinase
ICAM-1: intercellular adhesion molecule 1
CMC-I: carpometacarpal-I
NODs: nucleotide-binding oligomerization domains
NALP: Nacht domain-containing, leucine-rich repeat-containing and pyrin domain-containing protein
ASC: C-terminal caspase recruitment domain
AGEs: advanced glycation end-products
PPARγ: peroxisome proliferator-activated receptor γ
PGD_2_: prostaglandin D2
CXCL-1: chemokine (C-X-C motif) ligand 1
Cox-2: cyclooxygenase 2
mAb: monoclonal antibody
sRAGE: soluble RAGE.
